# Editorial: Heritage languages at the crossroads: cultural contexts, individual differences, and methodologies

**DOI:** 10.3389/fpsyg.2025.1553397

**Published:** 2025-01-28

**Authors:** Brechje van Osch, Selim Tiryakiol, Nadine Kolb, Alicia Luque, Jason Rothman

**Affiliations:** ^1^Department of Language and Culture, UiT The Arctic University of Norway, Tromsø, Norway; ^2^Department of Primary Education, Istanbul Medeniyet University, Istanbul, Türkiye; ^3^Department of Cultural Studies and Languages, University of Stavanger, Stavanger, Norway; ^4^Department of Applied Language Studies, Nebrija University, Madrid, Spain; ^5^Nebrija Research Center in Cognition, Nebrija University, Madrid, Spain; ^6^Department of Linguistics and English Language, Lancaster University, Lancaster, United Kingdom

**Keywords:** heritage bilingualism, methodology, individual differences, cultural contexts, editorial

## Introduction

The field of heritage language (HL) bilingualism research began to take shape around three decades ago, driven by the realization that heritage speakers (HSs) possess distinct linguistic characteristics that set them apart from other types of language users, native and non-native, monolingual or bilingual alike. For our purposes, a HL is a minority language spoken at home in an otherwise “other” majority language context (Rothman, 2009). In this context, the majority societal language(s) (SL(s)) is(are) omnipresent, while the HL is restricted to the home or a smaller community, typically resulting in reduced input and opportunities for HL use, and resulting in highly variable outcomes in individual HL speakers. Despite both being natives of the HL in focus (Rothman and Treffers-Daller, 2014), early HL research primarily focused on if/how HSs diverged linguistically by comparing them to L1-dominant users who grew up in the homeland. In recent years, HL research focus has shifted in various respects. First, there has been growing criticism of the default use of so-called monolingual control groups in bilingualism research in general. Simply put, the argument is that non-bilingual comparison groups are not theoretically, methodologically or ecologically appropriate for many—not all—questions asked in the contemporary theoretical landscape, nor are they reflective of today's interconnected world (Rothman et al., 2023; Wiese et al., 2022). Bilingual comparison groups have been proposed as a more suitable alternative for many research questions. Related to this, there is increasing advocacy for a more holistic approach to multilingualism research that takes into account all languages spoken by bilinguals, rather than focusing exclusively on one language (De Houwer, 2023). Furthermore, it has become increasingly clear that HSs are not a monolithic entity. Rather, the label HS represents a wide spectrum of linguistic profiles and outcomes. Consequently, more linguistic acquisition and processing studies have begun to explore individual differences and the sociolinguistic variables—at both the individual and the community level—that can explain HL continua. Lastly, answering calls in the previous literature (Bayram et al., 2020), online methods, including neuroimaging, that measure language processing in real time are becoming more widely used in the study of HL bilingualism (e.g., Fuchs, 2022; Hao et al., 2024; Jegerski, 2018; Keating et al., 2016; Keating, 2024; Luque et al., 2023).

This Research Topic sought to encourage continued exploration in these directions, emphasizing three main focal points, as indicated by the title: cultural contexts, individual differences, and methodologies. Each of these focal points will be discussed briefly, followed vis-à-vis an examination of how the papers in this Research Topic contribute to these themes.

## Cultural contexts

The field of (psycho-)linguistics has traditionally centered on understanding how languages are represented and processed in the minds of individual speakers. However, any such emphasis on the individual runs the risk of overlooking deterministic variables—particularly important for certain questions—such as the fact that language is also a shared construct within communities, shaped by social and cultural factors. Recently, there has been increasing recognition of the intricate relationship between language, culture, and society, a trend reflected in several papers in this Research Topic.

Wang and Li explore the stratifications and power dynamics among different Chinese HLs in Sydney. Their findings reveal that while parents prioritize Mandarin due to its perceived profitability compared to other Chinese dialects, English remains the most prestigious language in their eyes. Parafita Couto et al. focus on code-switching among Papiamento-Dutch HSs in the Netherlands. While their study primarily investigates the linguistic constraints on code-switching, they also uncover a preference for switching from Papiamento to Dutch rather than the reverse, reflecting what they claim to be the social dynamics within the community. Warditz and Meir's research investigates language attitudes among Ukrainian-Russian bilinguals who migrated to Austria or Germany, showing that while Ukrainian has gained symbolic value, Russian retains practical utility despite its negative symbolic status. Cruz examines the frequency, context, and pragmatic functions of diminutives in heritage Spanish in Southern Arizona using corpus data, and reports that these forms are used not merely as linguistic tools in this community, but also convey cultural meaning, playing a vital role in how speakers express their bilingual identity. Collectively, these studies underscore the crucial role of cultural and social contexts in bilingualism, showing that factors like language status, prestige, and speakers' identities and attitudes can vary significantly across contexts and greatly influence how languages are acquired and/or maintained.

## Individual differences

Historically, the field of psycholinguistics has often approached HSs as a uniform group. However, there is growing recognition that various groups of HSs, even within the same but especially across different language pairings and geographical contexts can present substantial differences and that individual differences among HSs even from the same context can be significant, sometimes even exceeding those between individual HSs and monolingual speakers (Kupisch and Rothman, 2018). Consequently, it is increasingly clear that HSs exist on a continuum, whereby by no one-size-fits-all approach can adequately apply. A key question, therefore, is which variables most significantly contribute to individual differences in outcomes. Several papers in this Research Topic address precisely this question.

Di Pisa et al. examine how HSs of Italian respond to subject-verb agreement errors, in both marked and unmarked contexts. The results show that HSs displayed greater sensitivity for marked features, and moreover, that the effect of markedness was more pronounced in speakers with higher proficiency, lower language use, and a higher age of onset of bilingualism. Grose-Hodge et al. examine school-aged HSs of Polish in the UK regarding their receptive and productive grammar, vocabulary, and fluency in both languages. They show that exposure, language aptitude, and motivation affect Polish proficiency, while only aptitude and age influence English. Böttcher and Zellers analyze filler particles in German and Russian as HLs and SLs using data from the RUEG corpus. Their findings revealed that the frequency and type of filler particle used varies by linguistic register as well as by speaker's age and gender.

These studies highlight the importance of a range of extra-linguistic variables, underscoring the crucial importance of either meticulously controlling for these factors or including them as covariates in analyses.

## Methodologies

Several articles in this Research Topic make significant methodological contributions. Fridman et al. employ network modeling (Freeborn et al., 2023), a technique relative new to linguistics. Compared to traditional methods, network modeling is more suitable for handling complex, dynamic, multivariate systems of interrelated variables, thus allowing researchers to gain a more comprehensive picture of the complexity of the bilingual experience. The authors recommend its broader application in future research.

Another methodological issue explored within this Research Topic concerns the reliability and validity of proficiency assessments. Luque et al. examined objective assessments, including the LexTale-Esp lexical decision task (Izura et al., 2014) and the “Modified DELE” (VGT; Montrul, 2005), alongside subjective assessments for Spanish HSs. While objective measures showed moderate to high internal reliability, their limited construct validity highlights challenges in capturing the multifaceted and ecologically valid nature of HL proficiency. Subjective assessments, by contrast, aligned more closely with real-world HL experiences, such as interactions with friends and self-talk, whereas objective measures correlated with compartmentalized family language use. These findings underscore the need for inclusive and ecologically valid approaches to account for the diverse and dynamic nature of Spanish HL proficiency. In a similar study, Hržica et al. examine Croatian-Italian bilinguals in Croatia, focusing on lexical diversity and syntactic complexity in both standard Italian and the Istrovenetian dialect. Their findings show modest correlations between objective measures and self-assessments for standard Italian, but stronger and more consistent correlations for Istrovenetian, suggesting participants have a more accurate perception of their proficiency in the dialect. These two studies highlight the complexity of assessing language proficiency in bilingual and diglossic communities, and emphasize the importance of integrating both objective and subjective measures for a more ecologically valid evaluation of bilingual proficiency.

Another methodological issue concerns the comparison of online and offline measures. Uygun investigates definiteness in heritage Turkish, finding that HSs are less accurate than monolinguals in offline judgments. However, in self-paced reading tasks, both groups exhibit similar sensitivity to definiteness and plurality. The findings of this study aligns with previous research that highlights the dissociation between offline and online measures in HS (Bayram et al., 2020), underscoring the necessity of data triangulation in this population.

Finally, Koronkiewicz and Delgado's contribution offers a methodological insight into the study of code-switching, showing that Spanish HSs' acceptance of code switched sentences is not differentially affected by whether the switched word is a cognates or a culturally specific item, suggesting that researchers can safely include such items in their studies.

## The interconnected dynamics of the HL and the SL

In addition to the primary themes explored in this Research Topic, an additional recurring one is the intricate relationship between the HL and the SL. Kan et al. investigate semantic knowledge in preschool HSs of Cantonese in the U.S., finding that, in a Word Association Task, children produce relatively more syntagmatic responses (contextual associations, such as “apple”—“eat”) in Cantonese than in English, along with a higher incidence of errors and language switches. The results also show a relationship between paradigmatic responses (i.e. based on shared categories or meaning, e.g. apple - fruit) in both languages, suggesting HL word representations may influence those in the second language (L2). Similarly, Casper et al. explore the interplay between HL Spanish and SL English in the processing of sentences with competing cues from both languages. Contrary to expectation, participants predominantly relied on agreement (the Spanish cue) in both languages. Additionally, higher proficiency in English correlated with faster reading times across languages, potentially due to the typological proximity of Spanish and English. Kim and Yim explore how literacy practices impact HL development in 4- to 5-year-old HSs of Korean in Australia. Through parental questionnaires and video analyses they show that parents employ different literacy strategies depending on the language. Moreover, a rich Korean literacy environment is found to positively impact both Korean and English language skills. In the related field of code-switching, Sedarous and Baptista show that English-dominant HSs of Arabic adhere to English movement constraints in their judgments of code-switched sentences with resumptive pronouns, in both code-switching directions. This suggests that these speakers converge on a single structural representation for code-switched speech.

The findings from the studies discussed in this paragraph underscore the intertwined nature of the various languages in the multilingual mind, underscoring that only focusing on one language may obscure the complete picture of the complexity and dynamics of multilingual competence.

## Revisiting the role of age of onset: HSs vs. L2 learners

A final issue explored within this Research Topic revisits the long-standing debate as to whether an early onset of language acquisition provides HSs with an advantage over L2 learners (Montrul, 2012). Two papers in this Research Topic address this question. Ge et al. investigated whether phonological advantages in HSs extend to novel word learning. Focusing on Mandarin, they show that HSs perform similarly to L2 speakers; both groups show a learning effect in segmental conditions (consonants and vowels), but struggle to utilize lexical tone–a suprasegmental feature for distinguishing between minimal pairs. Similarly, Prela et al. compare HSs and L2 learners of Greek regarding targeting several grammatical properties in English and Greek. Their results indicate that HSs of Greek do not outperform their L2 counterparts; in fact, the latter displayed more native-like and less variable performance.

While these two studies challenge the widely adopted notion that an early onset of acquisition confers advantages in language acquisition (Bley-Vroman, 1990), in the greater context of various literatures that seek to address this same question (e.g., studies comparing child L2 acquisition to adult L2 acquisition as well as other studies withe HS to L2 group comparisons), this question is far from settled and is likely to continue engaging researchers for years to come.

Taken together, these contributions collectively highlight the multifaceted nature of heritage language research, offering valuable insights that bridge cultural, individual, and methodological dimensions, while paving the way for future inquiries into the dynamic interplay of multilingualism and its broader social and cognitive contexts.
